# Does discontinuation of antithrombotics affect the diagnostic yield of small bowel capsule endoscopy in patients demonstrating obscure gastrointestinal bleeding?

**DOI:** 10.3164/jcbn.17-142

**Published:** 2018-04-03

**Authors:** Shiro Nakamura, Toshio Watanabe, Sunao Shimada, Yuji Nadatani, Koji Otani, Tetsuya Tanigawa, Takako Miyazaki, Masaki Iimuro, Yasuhiro Fujiwara

**Affiliations:** 1 Department of Inflammatory Bowel Disease, Division of Internal Medicine, Hyogo College of Medicine, 1-1 Mukogawa-cho, Nishinomiya City, Hyogo 663-8501, Japan; 2 Department of Gastroenterology, Osaka City University Graduate School of Medicine, 1-4-3 Asahimachi, Abeno-ku, Osaka 545-8585, Japan

**Keywords:** small intestine, capsule endoscopy, antithrombotics, obscure gastrointestinal bleeding

## Abstract

A large proportion of patients demonstrating obscure gastrointestinal bleeding (OGIB) are antithrombotic users and need to undergo small bowel capsule endoscopy (SBCE). We examined the effect of discontinuation of antithrombotics on the diagnostic yield of SBCE. Additionally, we assessed predictive factors associated with positive SBCE findings. Our study included 130 patients using antithrombotics who underwent SBCE for overt OGIB. The primary endpoint was the difference in the rate of positive SBCE findings between patients who continued and those who discontinued antithrombotics. Secondary endpoints were to investigate the effect of discontinuation of antithrombotics using a propensity score analysis, and to assess predictive factors associated with a positive SBCE. Among the 73 patients who continued use of antithrombotics, 36 (49.3%) patients demonstrated positive findings, while among the 57 patients who discontinued antithrombotics, 35 (61.4%) patients showed positive findings. Rates of positive SBCE findings didn’t differ between the two groups. After we performed propensity score matching, discontinuation didn’t affect the rate of positive SBCE findings. The lowest hemoglobin level was the only independent predictive factor associated with positive SBCE findings. In conclusion, discontinuation of antithrombotic therapy didn’t affect the diagnostic yield of SBCE in patients presenting with overt OGIB.

## Introduction

Obscure gastrointestinal bleeding (OGIB) is defined as persistent or recurrent bleeding associated with negative findings on upper and lower gastrointestinal (GI) endoscopic evaluations.^(1)^ OGIB is noted in approximately 5% of patients presenting with GI bleeding of any type.^(2)^ Following the introduction of small bowel capsule endoscopy (SBCE) and double-balloon enteroscopy (DBE) in the early 21st century,^(3,4)^ the source of bleeding was detected in the small intestine in a majority of OGIB patients.^(5,6)^ Although SBCE and DBE show a comparable diagnostic yield in small-bowel diseases including OGIB, and DBE is superior to SBCE in terms of endoscopic treatment including biopsies, hemostasis, polypectomy, and dilation,^(6)^ SBCE, owing to its noninvasiveness, has become the first-line modality for the diagnosis of OGIB.^(7)^

Use of antithrombotics including antiplatelet agents and anticoagulants is associated with upper and lower GI bleeding.^(8–10)^ Recent studies revealed that users of antithrombotics constitute a large proportion of OGIB patients who require examination of the small intestine using an SBCE,^(5,11)^ and OGIB patients using antiplatelet agents and anticoagulants are more likely to show positive findings on SBCE examination than those not administered such agents.^(11,12)^

It should be noted that some patients with overt OGIB discontinue use of antithrombotics at the time of SBCE, which may affect endoscopic findings. Therefore, we examined the effect of discontinuation of antithrombotic therapy on the diagnostic yield of SBCE. We additionally assessed predictive factors associated with positive SBCE findings in patients using antithrombotics who develop overt OGIB.

## Materials and Methods

### Study design

We performed a retrospective analysis to investigate the effects of discontinuation of antithrombotics (antiplatelet agents or anticoagulants) on the diagnostic yield of SBCE in patients with overt OGIB. We enrolled 130 consecutive patients (75 men, mean age 71.9 years) using antithrombotics who underwent SBCE for overt OGIB at the Hospital of Hyogo College of Medicine (Nishinomiya, Japan) and Osaka City University Hospital (Osaka, Japan) between March 2004 and December 2015. We excluded OGIB patients who underwent DBE prior to undergoing an SBCE. OGIB was defined as overt bleeding (hematemesis, hematochezia, or melena) or occult bleeding (positive fecal occult blood test, iron deficiency anemia, or an acute drop in hemoglobin) in a patient with no pathological causes identified using conventional endoscopic techniques including an esophagogastroduodenoscopy and colonoscopy.^(13)^ Patients demonstrating occult OGIB were excluded from this study. The study protocol conformed to the principles of the Declaration of Helsinki and was approved by the Institutional Review Board of Hyogo College of Medicine and the Osaka City University Graduate School of Medicine. Written informed consent was obtained from all participants prior to the examination.

### SBCE procedure and data interpretation

SBCE was performed using a PillCam SB1 or SB2 (Given Imaging, Ltd., Yoqneam, Israel). Patients fasted for 12 h before swallowing the capsule. Fluids were allowed 2 h later, followed by a light meal after another 2 h. Data were collected for up to 8 h after capsule ingestion. After 8 h, the sensor array and recording device were removed. The SBCE digital image stream was reviewed and interpreted independently by 2 endoscopists (TW and SS) who were blind to treatment administered to patients. Findings including mucosal breaks (ulcer or erosion), telangiectasia, tumors, and blood pooling without definite lesions to explain the bleeding, were considered positive, while other lesions with a low probability of bleeding, viz., isolated red spots erythema, and small polyps were considered negative.^(14)^ The culprit lesion was defined as one showing any of the positive findings. In cases demonstrating multiple positive findings on SBCE examination, a single primary finding considered the most indicative of GI bleeding was recorded. If endoscopic findings differed between the two interpreters, the findings were reviewed together by both blinded interpreters to reach a consensus.

### Endpoints

The primary endpoint was the difference in the rate of positive SBCE findings between patients who continued and those who discontinued use of antithrombotics. Secondary endpoints were to investigate the effect of discontinuation of use of antithrombotics on the diagnostic yield of SBCE using a propensity score analysis and to assess the predictive factors associated with positive SBCE findings using multiple logistic regression.

### Statistical analysis

In this study, patients with overt OGIB using antithrombotics were divided into a continuation and discontinuation group, and we compared the difference in the diagnostic yield of SBCE between the two groups. We defined discontinuation as stopping use of all antithrombotics for more than a day at the time of SBCE examination. A list of baseline clinical variables was specified for the assessment of predictive factors associated with positive SBCE results. Data are presented as mean ± SD for continuous variables and as numbers (percentages) for categorical variables. For categorical data, comparisons between groups were performed using the chi-squared test (or the Fisher exact test when necessary because of small sample sizes), whereas continuous data were compared using the Student’s *t* test. Effects of clinical variables on positive SBCE results were estimated by calculating odds ratios (ORs) and 95% confidence intervals (CIs) using logistic regression analyses. Factors showing a *p* value <0.2 using univariate logistic analyses were included in subsequent multivariate logistic regression analyses.

Furthermore, a propensity score-matched cohort was created by attempting to match each patient who underwent SBCE after discontinuation of antithrombotics with a patient who underwent the procedure without discontinuation of antithrombotics. We performed a 1:1 match using a matching technique to reduce the effects of discontinuation-selection bias and potential confounding factors between each group.^(15,16)^ We chose 11 variables that could possibly influence the outcomes (positive SBCE findings) to generate a propensity score ranging between 0 and 1 using logistic regression. These variables included age, gender, drinking and smoking habits, lowest hemoglobin (Hb) level, use of nonsteroidal anti-inflammatory drugs (NSAIDs), intake of proton pump inhibitors (PPIs), histamine-2-receptor antagonists (H_2_RAs), corticosteroids, and prostaglandin E_1_ derivatives, and the Charlson comorbidity index (CCI)—an index that includes 19 selected medical conditions, all related to an increased risk of mortality.^(17)^ Absolute standardized differences (ASD) were computed to evaluate matching effectiveness. Statistical analyses were performed using SPSS software ver. 21.0 for Windows (SPSS Inc., Tokyo, Japan) and the R statistical package (ver. 2.13.0; free download from http://www.r-project.org, Access date February 23, 2016). All statistical tests were 2-sided, and *p* values <0.05 were considered statistically significant.

## Results

### Baseline characteristics of patients

Among the 130 patients presenting with overt OGIB who underwent SBCE, 73 patients continued use of antithrombotics, while 57 patients discontinued use of these agents. The time points of discontinuation were as follows: 1–2 days (*n* = 11), 3–6 days (*n* = 8), and 7 days≤ (*n* = 38). Anticoagulants were administered to 32 patients (43.8%) in the continuation group and 19 patients (33.3%) in the discontinuation group, while 59 patients (80.8%) in the continuation group and 47 patients (82.5%) in the discontinuation group received antiplatelet therapy. Table 1 shows baseline demographic information, as well as clinical variables. Compared to overt OGIB patients who discontinued use of antithrombotics, overt OGIB patients who continued use of these agents demonstrated a higher rate of concomitant use of H_2_RA, with a tendency to be higher in the lowest Hb level and to use prostaglandin E_1_ derivatives more frequently. The rate of concomitant PPI use and mean CCI were comparable between the two groups.

### Primary endpoint

Among the 73 patients who continued use of antithrombotics, 36 (49.3%) patients showed positive findings in the small intestine [ulcers/erosions (*n* = 24), telangiectasias (*n* = 7), tumors (*n* = 4), and blood pooling (*n* = 1)], while among the 57 patients who discontinued use of these agents, 35 (61.4%) patients showed positive findings [ulcers/erosions (*n* = 17), telangiectasias (*n* = 11), tumors (*n* = 3), and blood pooling (*n* = 4)]. The diagnostic yield of SBCE did not differ between the two groups (Table 2).

### Secondary endpoints

Following propensity score matching, we obtained 53 matched pairs of patients who continued or discontinued antithrombotics. Baseline characteristics of the two groups were comparable, and there were no significant differences in baseline characteristics of the 53 matched pairs (Table 1). As shown in Table 2, even after propensity score matching, the diagnostic yield did not differ between the continuation and discontinuation group.

To further confirm the effect of discontinuation of use of antithrombotics on the diagnostic yield of SBCE, we excluded patients who had discontinued antithrombotic therapy for <3 days (*n* = 11), and then propensity score matching analysis was performed. The diagnostic yield of SBCE did not differ between the two groups: the diagnostic yield in the continuation and discontinuation group was 41.3% and 56.5%, respectively (*n* = 46 for each group).

Next, we evaluated predictive factors associated with positive SBCE findings in overt OGIB patients who used antithrombotics. The lowest Hb level prior to the SBCE examination was associated with positive SBCE findings as noted using univariate analysis with a *p* value = 0.05 (OR 1.164 per 1 g/dl decrease, 95% CI 1.00–1.355), whereas other variables including age, NSAID use, and discontinuation of antithrombotics did not show an association with positive findings (Table 3). Multivariate analysis showed the lowest Hb level was the only independent predictive factor associated with positive SBCE findings (OR 1.189 per 1 g/dl decrease, 95% CI 1.012–1.397, Table 4).

## Discussion

Actively bleeding lesions are considered likely to be detected using SBCE, even though detailed observation of the lesion might not be possible due to bleeding. Thus, it could be hypothesized that discontinuation of antithrombotic therapy may reduce the diagnostic yield of SBCE, because discontinuing use of such agents could result in cessation of bleeding. However, in this study, the rate of positive SBCE findings in patients who discontinued use of antithrombotics was not low, and was in fact slightly higher than in those who continued use of these agents, although the difference between the two groups was not statistically significant. Because patients who discontinued use of antithrombotics might have severe lesions, which can be easily detected using SBCE, we performed propensity score matching analysis to reduce the effects of discontinuation-selection bias, which confirmed similar results. Our data thus suggest that discontinuation of antithrombotics does not affect the diagnostic yield of SBCE in patients with overt OGIB, and this information would be very relevant to clinical practice.

In this study, intestinal mucosal breaks (ulcers or erosions) was the leading cause of bleeding in both, the continuation and discontinuation group, followed by telangiectasias, which was the most common finding in other some studies.^(12,14,18)^ This difference could be explained by the fact that more than 70% of our OGIB patients in both groups reported intake of low-dose aspirin (LDA). We have previously reported that LDA users who develop peptic ulcers additionally demonstrate a high incidence of small intestinal mucosal breaks.^(19)^ The strong ulcerogenic effect of LDA on the small intestine has been confirmed by prospective studies that involved healthy subjects.^(20,21)^ Although LDA-induced intestinal mucosal breaks rarely heal spontaneously while LDA therapy continues,^(19,20)^ it is unclear whether discontinuation of LDA therapy leads to healing of such lesions. We found that among 57 overt OGIB patients who discontinued use of antithrombotics, 17 patients (29.8%) demonstrated mucosal breaks suggesting that LDA-induced mucosal breaks require some time to heal even after discontinuation of LDA therapy.

Previous studies have reported that several factors, viz., age, sex, overt bleeding,^(21)^ low Hb level, early SBCE examination after the last bleeding episode,^(22)^ and presence of comorbidities may affect the diagnostic yield of SBCE in OGIB patients,^(23)^ but no study has focused on predictive factors associated with positive SBCE findings in OGIB patients using antithrombotics. Because use of antithrombotics is a major cause of OGIB, evaluation of predictive factors associated with positive SBCE findings in users of antithrombotics is considered clinically important. To the best of our knowledge, our study is the first to identify the lowest Hb level obtained prior to an SBCE examination as an independent predictor associated with positive SBCE findings in overt OGIB patients using antithrombotics. There is no clarity regarding the association between the lowest Hb level and positive findings observed on SBCE examination. A possible explanation could be that the lowest Hb level may be a surrogate marker of the severity of intestinal lesions, which could influence the SBCE result.

Limitations of our study are: 1) Ours was a retrospective study involving a relatively small number of patients. Large-scale studies in which more than 500 OGIB patients were recruited have been performed to evaluate the predictive factors associated with positive SBCE findings.^(21,24)^ However, these studies included both overt and occult OGIB patients and were not restricted to users of antithrombotics. Ours is one of the largest studies ever performed on overt OGIB patients who reported use of antithrombotics. Furthermore, we performed propensity score matching analysis to reduce the effects of discontinuation-selection bias (which could occur in a retrospective study like ours), thereby confirming that discontinuing use of antithrombotics did not affect the diagnostic yield of SBCE. 2) Duration of discontinuation of antithrombotics was <1 week in 19 patients (33.3%) belonging to the discontinuation group. Therefore, the effect of discontinuation of these agents on the SBCE results might not be conclusively and completely expressed in such patients. 3) We cannot deny the possibility that lesions detected by SBCE examination were not the true source of GI bleeding. However, such misinterpretation pertaining to culprit lesions for overt OGIB, if any, would be equally distributed between the continuation and discontinuation groups. Thus, this factor would have a minor effect, if any, on our results.

In conclusion, discontinuation of antithrombotics did not affect the diagnostic yield of SBCE in patients with overt OGIB, and the lowest Hb level was a predictive factor associated with positive SBCE findings. Therefore, patients showing a low Hb level who develop overt OGIB during treatment with antithrombotics should undergo SBCE regardless of continuation or discontinuation of these drugs.

## Figures and Tables

**Table 1:** Baseline characteristics and propensity score matched baseline characteristics

	Baseline				Propensity score matched baseline			
	Continuation group (*n* = 73)	Discontinuation group (*n* = 57)	*p*		Continuation group (*n* = 53)	Discontinuation group (*n* = 53)	*p*	ASD (%)
Age (years)	70.9 ± 9.2	73.1 ± 9.2	0.188		72.3 ± 8.9	72.5 ± 9.1	0.88	3
Male	44 (60.3%)	31 (46.3%)	0.097		58	29 (54.7%)	1	0
Smoking habit	12 (16.4%)	5 (7.5%)	0.172		10	5 (9.4%)	1	0
Alcohol intake	12 (16.4%)	11 (16.4%)	0.997		18	10 (18.9%)	0.609	10.1
Lowest Hb level (g/dl)	8.1 ± 2.5	7.5 ± 2.3	0.16		7.8 ± 2.3	7.7 ± 2.3	0.621	9.7
Concomitant agent								
NSAIDs	12 (16.4%)	11 (16.4%)	0.997		16	8 (15.1%)	1	0
PPI	34 (46.6%)	31 (46.3%)	0.971		56	28 (52.8%)	1	0
H_2_RA	19 (26.0%)	6 (9.0%)	0.016		12	6 (11.3%)	1	0
Corticosteroid	2 (2.7%)	4 (5.9%)	0.6		4	2 (3.8%)	1	0
Prostaglandin E_1_ analogue	4 (5.5%)	0 (0.0%)	0.151		0	0 (0.0%)	1	0
CCI	1.82 ± 1.49	1.63 ± 1.49	0.476		1.64 ± 1.52	1.55 ± 1.48	0.529	12.4
Anticoagulant agent	32 (43.8%)	19 (33.3%)	0.223		14 (26.4%)	19 (35.8%)	0.327	19.2
Warfarin	30 (41.1%)	18 (31.6%)	0.265		14 (26.4%)	19 (35.8%)	0.327	19.2
DOACs	2 (2.7%)	1 (1.8%)	1		0 (0.0%)	0 (0.0%)	1	0
Antiplatelet agent	59 (80.8%)	47 (82.5%)	0.812		43 (81.1%)	43 (81.1%)	1	0
Low-dose aspirin	54 (74.0%)	40 (70.2%)	0.631		39 (73.6%)	38 (71.7%)	0.83	4.2
P_2_Y_12_ receptor inhibitors	20 (27.4%)	11 (19.3%)	0.282		12 (22.6%)	11 (20.8%)	0.816	4.6
Cilostazol	4 (5.5%)	3 (5.3%)	1		3 (5.7%)	3 (5.7%)	1	0
Duration of discontinuation (days)								
1–2		11 (19.3%)				10 (18.9%)		
3–6		8 (14.0%)				7 (13.2%)		
≥7		38 (66.7%)				36 (67.9%)		

ASD, Absolute standardized difference; Hb, hemoblobin; NSAID, nonsteroidal anti-inflammatory drug; PPI, proton pump inhibitors; H_2_RA, histamine-2-receptor antagonist; CCI. Charlson comorbidity index; DOAC, direct oral anticoagulant.

**Table 2:** Comparison of the diagnostic yield of SBCE between over OGIB patients who continued and who discontinued antithrombotic agents

	Continuation group	Discontinuation group	*p*
Before propensity score matching			
Number of patients	73	57	
Diagnostic yield	36 (49.3%)	35 (61.4%)	0.169
Culprit lesion			
Blood pooling only	1 (1.4%)	4 (7.0%)	
Ulcer/erosion	24 (32.9%)	17 (29.8%)	
Telangioectasia	7 (9.6%)	11 (19.3%)	
Tumor	4 (5.5%)	3 (5.3%)	

After propensity score matching			
Number of patients	53	53	
Diagnostic yield	26 (49.1%)	32 (60.4%)	0.242
Culprit lesion			
Blood pooling only	1 (1.9%)	2 (3.8%)	
Ulcer/erosion	16 (30.2%)	16 (30.2%)	
Telangioectasia	5 (9.4%)	11 (20.8%)	
Tumor	4 (7.5%)	3 (5.7%)	

SBCE, small bowel capsule endoscopy; OGIB, obscure gastrointestinal bleeding.

**Table 3:** Univariate analysis of predictive factors associated with positive SBCE findings in overt OGIB patients on antithrombotic agents

Variables	Reference	OR	95% CI	*p*
Age (per 1 year increase)		1.014	0.977–1.052	0.472
Male	Female	1.296	0.644–2.607	0.468
Hemoglobin level (per 1 g/dl decrease)		1.164	1.000–1.355	0.05
Smoking habit	Non-smoking	2.197	0.726–6.643	0.163
Alcohol intake	No intake	0.888	0.360–2.188	0.796
NSAID use	Non-user	1.098	0.443–2.723	0.84
antiplatelet use	Non-user	1.53	0.646–3.630	
Anticoagulant use	Non-user	1.007	0.499–2.033	0.985
PPI use	Non-user	1.755	0.874–3.525	0.114
Corticosteroid use	Non-user	0.399	0.070–2.257	0.298
Discontinuation of antithrombotics	Continuation	1.635	0.809–3.304	0.171
CCI ≥5	CCI <5	3.117	0.622–15.618	0.167

SBCE, small bowel capsule endoscopy; OGIB, obscure gastrointestinal bleeding; OR, odds ratio; CI, confidence interval; NSAID, nonsteroidal anti-inflammatory drug; PPI, proton pump inhibitors; CCI, Charlson comorbidity index.

**Table 4:** Multivariate analysis of predictive factors associated with positive SBCE findings in overt OGIB patients on antithrombotic agents

Variables	Reference	OR	95% CI	*p*
Hemoglobin level (per 1 g/dl decrease)		1.189	1.012–1.397	0.035
Smoking habit	Non-smoking	3.262	0.97–10.914	0.055
PPI use	Non-user	1.62	0.773–3.395	0.201
Discontinuation of antithrombotics	Continuation	1.579	0.749–3.326	0.23
CCI ≥5	CCI <5	2.518	0.480–13.210	0.275

SBCE, small bowel capsule endoscopy; OGIB, obscure gastrointestinal bleeding; OR, odds ratio; CI, confidence interval; PPI, proton pump inhibitors; CCI, Charlson comorbidity index.

## Abbreviations

ASD: absolute standardized difference
CCI: Charlson comorbidity index
CI: confidence interval
DBE: double-balloon enteroscopy
GI: gastrointestinal
Hb: hemoglobin
H_2_RA: histamine-2-receptor antagonist
LDA: low-dose aspirin
NSAID: nonsteroidal anti-inflammatory drug
OGIB: obscure gastrointestinal bleeding
OR: odds ratio
PPI: proton pump inhibitor
SBCE: small bowel capsule endoscopy
